# Are Flavonoids Effective Antioxidants in Plants? Twenty Years of Our Investigation

**DOI:** 10.3390/antiox9111098

**Published:** 2020-11-09

**Authors:** Giovanni Agati, Cecilia Brunetti, Alessio Fini, Antonella Gori, Lucia Guidi, Marco Landi, Federico Sebastiani, Massimiliano Tattini

**Affiliations:** 1 Institute of Applied Physics ‘Carrara’, National Research Council of Italy (CNR), Via Madonna del Piano 10, Sesto F.no, I-50019 Florence, Italy; g.agati@ifac.cnr.it; 2 Institute for Sustainable Plant Protection, National Research Council of Italy (CNR), Via Madonna del Piano 10, I-50019, Sesto F.no, Florence, Italy; cecilia.brunetti@ipsp.cnr.it (C.B.); federico.sebastiani@ipsp.cnr.it (F.S.); 3 Department of Agriculural and Environmental Sciences - Production, Landscape, Agroenergy, University of Milan, Via Celoria 2, I-20133 Milan, Italy; alessio.fini@unimi.it; 4 Department of Agriculture, Food, Environment and Forestry, University of Florence, Viale delle Idee 30, Sesto F.no, I-50019 Florence, Italy; antonella.gori@unifi.it; 5 Department of Agriculture, Food and Environment, University of Pisa, Via del Borghetto 80, I-56124 Pisa, Italy; lucia.guidi@unipi.it (L.G.); marco.landi@unipi.it (M.L.)

**Keywords:** antioxidant enzymes, cytoplasm-located flavonoids, early land plants, flavonols, hydrogen peroxide, photoprotection, reactive oxygen species, UV-B radiation, vacuolar flavonoids

## Abstract

Whether flavonoids play significant antioxidant roles in plants challenged by photooxidative stress of different origin has been largely debated over the last few decades. A critical review of the pertinent literature and our experimentation as well, based on a free-of-scale approach, support an important antioxidant function served by flavonoids in plants exposed to a wide range of environmental stressors, the significance of which increases with the severity of stress. On the other side, some questions need conclusive answers when the putative antioxidant functions of plant flavonoids are examined at the level of both the whole-cell and cellular organelles. This partly depends upon a conclusive, robust, and unbiased definition of “a plant antioxidant”, which is still missing, and the need of considering the subcellular re-organization that occurs in plant cells in response to severe stress conditions. This likely makes our deterministic-based approach unsuitable to unveil the relevance of flavonoids as antioxidants in extremely complex biological systems, such as a plant cell exposed to an ever-changing stressful environment. This still poses open questions about how to measure the occurred antioxidant action of flavonoids. Our reasoning also evidences the need of contemporarily evaluating the changes in key primary and secondary components of the antioxidant defense network imposed by stress events of increasing severity to properly estimate the relevance of the antioxidant functions of flavonoids in an in planta situation. In turn, this calls for an in-depth analysis of the sub-cellular distribution of primary and secondary antioxidants to solve this still intricate matter.

## 1. Introduction

It has been known for decades that UV, particularly UV-B radiation, triggers the biosynthesis of flavonoids (for recent review articles see [1,2,3]), the vast class of phenylpropanoids comprising more than 8000 structures with a huge array of decorations [3]. The flavonoid metabolic pathway was raised during the water-to-land transition of plants, when plants were challenged against an abrupt increase in UV-B radiation [3,4]. This led to the hypothesis that flavonoids have an almost exclusive UV-B screening role in photoprotection [5,6]. It was suggested that flavonoids have the peculiar capacity to absorb strongly over the UV-B portion of the solar spectrum, while offering negligible shielding to visible light radiation, thereby protecting DNA [7,8] and PSII [9,10] from damage. Nonetheless, the very same UV-B absorbing features belong to the vast majority of phenylpropanoids; a wide range of polyphenols display even greater capacity to shield sensitive leaf targets from high UV-B irradiance compared to flavonoids [11]. It has been inferred, therefore, that unveiling how and how much flavonoids serve photoprotective functions in the network of UV-screening phenylpropanoids synthesized by current-day land plants needs deeper analysis [12].

**Figure 1:**
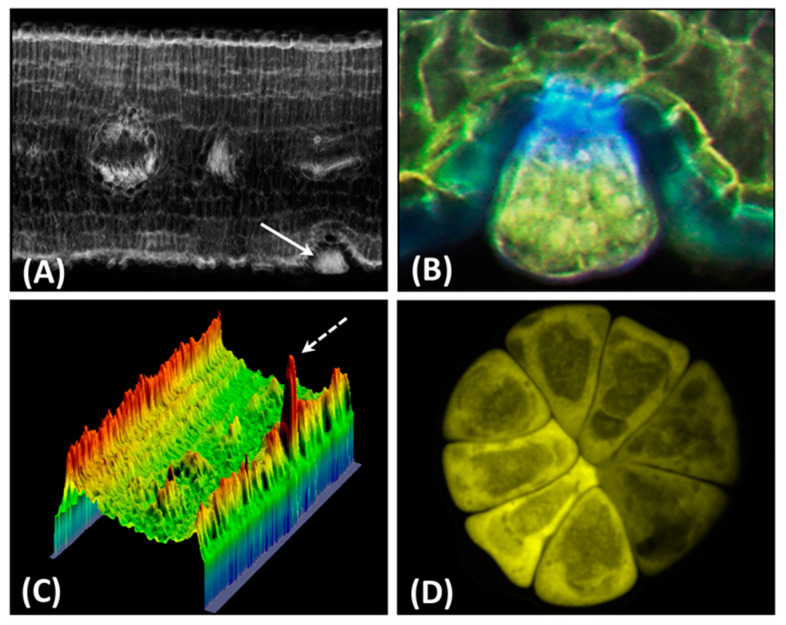
Flavonoids wxcluively accumulate in *P. latifolia* leaves. (**A**) Imaging of the ratio of fluorescence intensity at 580 nm (F580, flavonoid fluorescence) to fluorescence at 470 nm (F470, hydroxycinnamate fluorescence) of a Naturstoff-stained cross-section excited at 365 nm. (**B**) and (**D**) False-colour F580 images of the glandular trichome (denoted by the arrow in **A**), assigning the yellow channel to F580. (**C**) Three-dimensional false colour image of the cross section in (**A**). Highest values of F580/F470 in the glandular trichome (white arrows in A and C) indicates hydroxycinnamates do not appreciably accumulate in this organ. Methodological details given in Agati et al. [14].

The first time we encountered flavonoids was when examining leaves of *Phillyrea latifolia* adapted to deep shade (5% of full solar irradiance) or full sunlight [13]. We observed that glandular trichomes, distributed on both the adaxial and the abaxial leaf surface in fully developed leaves, exclusively synthesized hydroxycinnamic acid (HCA) or flavonoid derivatives in the shade or in full sunlight, respectively. The almost exclusive distribution of flavonoids in glandular trichomes of leaves growing in full sunlight was confirmed later using multispectral fluorescence microimaging (Figure 1) [14]. These observations led to the hypothesis that flavonoids have functional roles beyond the absorption of UV, particularly of UV-B radiation in photoprotection. In fact, flavonoids (particularly the dihydroxy B-ring-substituted structure detected in our studies) are less effective than HCA in absorbing UV-B wavelengths [15,16]. In a series of successive experiments, we also observed a steep increase in the ratio of dihydroxy to monohydroxy B-ring-substituted flavonoids in response to high light irradiance, in the presence or in the absence of UV radiation [17,18,19,20]. Based on the relative light screening and antioxidant properties of mono- and dihydroxy B-ring-substituted flavonoids (reviewed in [16]), we hypothesized that flavonoids might serve major antioxidant functions in photoprotection. We conducted studies, at very different scale levels, to support this hypothesis over the last two decades. Here, we discuss the significance of flavonoids as antioxidants in plants and their relative contribution to the integrated and modular network of antioxidant defenses plants activate when exposed to photooxidative stress of increasing severity. The matter is still under hot debate, in part because an unequivocal definition of a plant antioxidant is still missing [21,22,23]. 

## 2. Major Antioxidant Functions of Flavonoids in Photoprotection: Free-of-Scale Evidence 

When plants moved from water to colonize land they were challenged by a wide range of “novel” environmental pressures, including, but not limited to, an abrupt increase in UV-B radiation [24,25,26,27,28]. It has been believed for decades that the rise of flavonoid metabolism aimed at equipping plants with a more effective UV-B screening shield as compared to algae. However, this does not seem to be exactly the case (for critical reviews, see [24,29,30,31,32]). First, a range of algal species synthesizes a variety of mycosporine-like amino acids, which are more effective than flavonoids in absorbing UV-B wavelengths [16]. Second, flavonoids have minimum molar extinction coefficients (ε) over the 290-315, UV-B portion of the solar spectrum. Third, in plant organs most exposed to UV-B radiation, the synthesis of hydroxycinnamic acid (HCA) derivative declines in favor of flavonoid biosynthesis [12,13], even though HCAs absorb UV-B more efficiently than flavonoids [16]. Fourth, the biosynthesis of quercetin (or dihydroxy B-ring-substituted flavonoids) is strongly favored with respect to kaempferol (or monohydroxy B-ring-substituted flavonoids) biosynthesis following exposure to UV-B radiation, though quercetin and kaempferol have similar UV-B screening efficiency. Finally, a series of relatively recent experiments have shown UV-B radiation is not a prerequisite [19,33,34], and blue light is more effective than UV-B light in stimulating flavonoid biosynthesis [35]. There is increasing evidence suggesting that during the water-to-land transition, water stress and excessive light including, but not limited to UV-B radiation, drove most molecular innovations enabling early plants to successfully colonize land [36,37,38,39,40]. 

**Figure 2:**
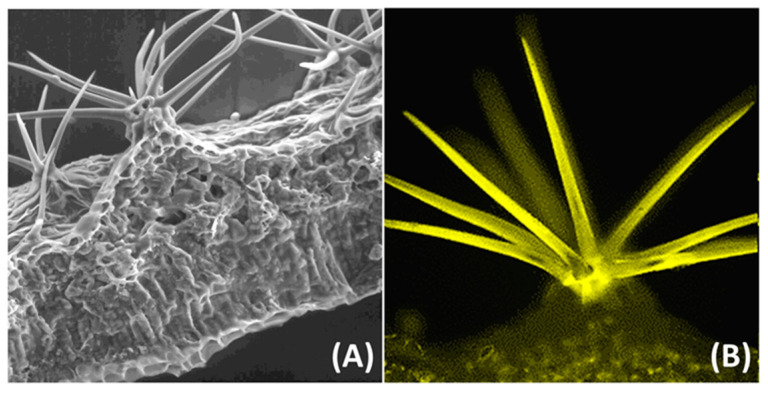
Mere UV-screening effects of flavonoids in non-glandular trichomes of *Cistus salvifolius* leaves. (**A**) View of a leaf cross-section in Cryo Scan Electron Microscopy (Cryo-SEM). (**B**) Fluorescence image recorded at 565-570 nm of cross-section stained with Naturstoff reagent, and excited at 488 nm. Flavonoids are coumaroyl derivatives of kaempferol 3-*O*-glucoside and are associated with the cell wall of the trichome arms, as detailed in Tattini et al. [42]. The occurrence of p-coumaric (max. absorbance at 315 nm) and kaempferol moieties (max absorbance at 351 nm) offers the best screening against UV-B and UV-A radiation.

Caldwell and colleagues [41] questioned the functional significance of flavonoids as mere UV-B absorbing pigments in UV-B exposed plants, approx. 40 years ago: they indeed noted that UV-B radiation promotes the biosynthesis of metabolites maximally absorbing over the UV-A portion of the solar spectrum. There are very few plant species synthesize flavonoids acylated with a range of HCA moieties, which may offer an effective shield against the penetration of the entire spectrum of UV wavelengths in the leaf (Figure 2).

**Figure 3:**
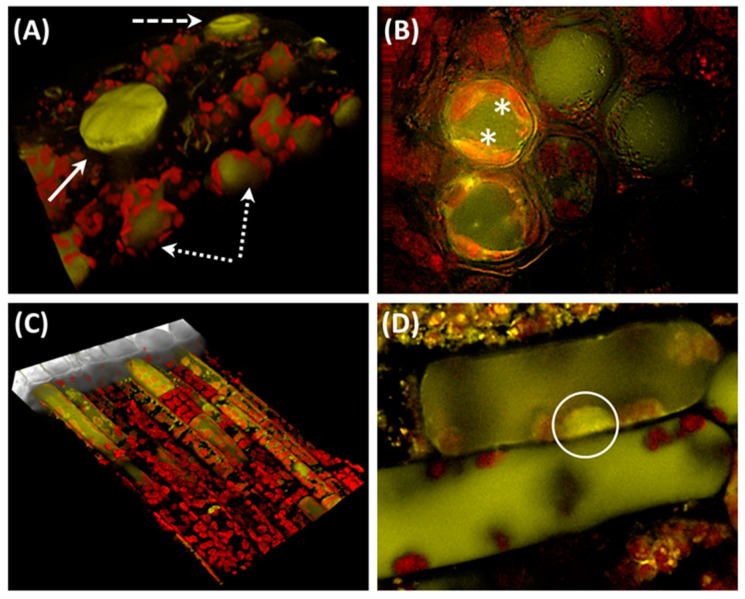
Flavonoids occur in different organs, cells and sub-cellular organelles. (**A**) Confocal laser scanning microscopy of *Ligustrum vulgare* leaves showing the accumulation of flavonoids in glandular trichomes (solid arrow), the vacuoles of mesophyll cells (dotted arrows) and the guard cells of stomata (*). (**B**) Flavonoids in mesophyll cells have not unique vacuolar accumulation, but are additionally associated to chloroplasts. (**C**) A 3D view of mesophyll cells: flavonoids accumulate in different layers of palisade tissue. (**D**) An enlarged view of flavonoid distribution in mesophyll cells, showing the location of flavonoids in the cell nucleus (enclosed in the circle). Tissue specimen were stained with Naturstoff reagent, excited at 488 nm and fluorescence recorded over both the 562–646 nm and the 687–757 nm waveband to detect flavonoids and chlorophyll, respectively. Pictures are merged images of F562-646 and F687-757.

We note that the vast majority of early studies examining the photoprotective functions of flavonoids exposed plants to unrealistic UV-B doses for very short time periods (e.g., see [43,44,45,46,47,48]). Consequently, flavonoids exclusively accumulated in leaf epidermal cells, further reinforcing the idea of their exclusive roles as UV-B screening pigments. We also observe that the concentration of UV-B absorbing compounds, as estimated through absorption at 300-330 nm of tissue extracts, was in some instances assumed to represent the flavonoid concentration [49,50,51]. This is actually wrong, as different phenylpropanoid classes effectively absorb over this portion of the solar spectrum. In other instances, identification of flavonoids was erroneously assessed based on the absorption spectra of isolated compounds [44]. Finally, while studies examining the UV-B responses of mutants lacking the biosynthesis of a range of flavonoids conclusively showed their essential roles against UV-B damage [44,51,52,53], they did not offer a conclusive explanation about how flavonoids afford photo-protection. It is logically hard to perform experiments with the aim of unveiling the molecular events that trigger the flavonoid biosynthesis and, at the same time of assessing their functional roles in plant-environment interactions: it is simply a matter of scale (scaling-up). Results arising from growth chamber experiments, in which model plant species are exposed to unrealistic light irradiance, should not be considered as informative to describe the complexity of plants growing in their natural habitats. High UV and visible light irradiance are just two of the range of concomitant environmental stressors plants face in the field [17,18]. The picture is complicated further by the observation that flavonoids occur in different plant organs, tissues and subcellular compartments in plants growing in full sun-exposed leaves (Figure 3; for a review see [31]). Exploring the functional roles of flavonoids in photoprotection is an ecophysiological, not only a “molecular biology” challenge. This, when coupled with our constitutive inability to leave well-traced paths, has made complex the study of the primary roles played by flavonoids in photoprotection.

Helen Stafford [29] hypothesized that a nascent and, hence, inefficient metabolism unlikely produced flavonoid at concentrations sufficient to provide an effective UV-B screening, thereby suggesting that flavonoids have alternative roles (i.e., developmental regulators) in response to UV-B stress. A few years later, Landry et al. [54] and Sheahan [15] first hypothesized for flavonoids an antioxidant, perhaps primary, function in UV-B treated leaves. As reported above, this hypothesis has been corroborated later by the steep increase in the ratio of dihydroxy to monohydroxy B-ring-substituted flavonoid glycosides in response to UV-B in a range of species [17,18,48,55,56,57,58]. The capacity to scavenge reactive oxygen species (ROS) of glycosylated flavonoids is mostly conferred by the catechol group in 3′-4′ position, since the most reactive and, hence, the most reducing OH-group in 3-position is preferentially glycosylated. In other words, quercetin, but not kaempferol derivatives, are effective antioxidants [17]. Interestingly, an increase in dihydroxy to monohydroxy B-ring-substituted flavonoids has been also reported in response to visible light [19,20], drought [17] and salinity stress (in the absence of UV radiation, [20]). A preferential increase in the concentration of the antioxidant quercetin glycosides was also shown in response to low temperature or nutrient deficiency in high light growing plants [59,60,61,62]. These observations are consistent with the notion of flavonoid biosynthesis being activated by over-reduction of photosynthetic electron transport chain (PETC), a common feature, together with ROS production plants face when exposed to different abiotic stressors [63,64]. Notably, that application of far-red light (700–780 nm), which is known to induce the oxidation of the PETC, depresses both the biosynthesis of flavonoids and the ratio of quercetin to kaempferol [65]. This supports the view the redox and/or ROS signals are key drivers for the biosynthesis of flavonols (and for the biosynthesis of other secondary metabolites as well [66,67,68,69]). This conforms to the notion that the activity of R2R3MYB proteins, regulating key genes of flavonol biosynthesis, is also under strict redox control, even because of posttranslational modifications [70,71,72,73,74].

It has been reasonably inferred, therefore, that the biosynthesis of flavonoids is activated by alteration in the redox potential of the cell and, in turn, flavonoids may help to limit oxidative damage primarily acting as antioxidants and not as light screeners in photoprotection [12,30,31]. These are likely ancestral functions of flavonoids, as early land plants underwent severe desiccation and large temperature fluctuations, in addition to high light intensity, when they moved from water [37,75]. In other words, “an excess of UV-B radiation” was just one (possibly not the most detrimental) of the “novel” environmental pressures plants faced when moving on land [25,28,76,77]. Early land plants had to live with and adapt to an ever-changing ROS environment from the very beginning [26]. The evolution of flavonoid (and the phenylpropanoid) metabolism as well as of TFs regulating their biosynthesis [4] represent key components of the suite of traits whose evolution has been responsible for the increased complexity of land plants, thereby allowing their radiation toward less favorable environments [4,5]. For example, in a range of modern land plants, a suite of flavonoid and hydroxycinnamate structures accumulate in plants growing under partial shading, with the monohydroxy B-ring-substituted structures dominating the phenylpropanoid pool [13,14,16]. These compounds are mostly distributed in the external leaf tissues, such as the epidermal cells and different types of trichomes (Figure 2), and may well represent a constitutively (i.e., in plants growing in partial shading) efficient shield against UV-B radiation [78,79]. The observation that monohydroxy B-ring-substituted phenylpropanoids are almost unresponsive, whereas dihydroxy B-ring-substituted flavones and flavonols increase in concentration because of UV-B, UV-A as well as visible light irradiance, offers strong support to a major antioxidant role of flavonoids in photoprotection [12,17,18,31]. We note indeed that photoprotection must be “measured” only when plants experience light irradiance largely exceeding that actually used for photosynthesis, i.e., under strong photoinhibitory conditions [80]. 

## 3. Do Flavonoids Play Relevant Antioxidant Functions in Plants? A Matter of Definition

The actual role of flavonoids as effective antioxidants in both plants and animals has been extensively debated over the last few decades [3,12,31,81,82,83]. Though flavonoids have great potential to counter the detrimental effects of the massive generation of reactive oxygen species (ROS), authoritative criticisms have been raised in many instances. Barry Halliwell, who provided a series of definitions of “antioxidants”, has always been critical of the effective ability of flavonoids to “delay, prevent or remove oxidative damage to a target molecule”, a widely accepted definition of antioxidant given in Gutteridge and Halliwell [21]. This is because the flavonoid structures with the greatest ability to reduce (i.e., flavonoid aglycones) the levels of oxidants are usually in very low concentrations in human tissues [84,85,86]. This, in turn, implies flavonoids cannot play systematic antioxidant functions. Even more, Halliwell [85], and Gutteridge and Halliwell [87] suggested that the beneficial effects of flavonoids on human health might be also due to their pro-oxidant actions, thereby activating a range of endogenous antioxidant defenses. The Gutteridge–Halliwell definition of antioxidant was shaped over time taking into account novel evidence from studies performed on humans, but has been largely used by plant biologists as well. Being wide in its nature, this definition is rather robust, since it applies to organisms in different kingdoms [88], and includes not only the substance ability to quench/scavenge oxidants, but also to limit the rate at which oxidants form, i.e., the substance ability to “avoid” oxidative stress. In other words, the way through which a substance “protects” a target molecule from oxidative damage does not matter. 

However, this has sparked and continues to spark debate among plant biologists. For instance, the capacity of flavonoids, both the colorless (e.g., flavones and flavonols) and the colored structures (i.e., anthocyanins) to strongly absorb over the UV- and the visible portion of the solar spectrum may effectively protect so-called sensitive targets from uncontrolled ROS-generation, and from the consequential oxidative damage [31,86,89]. Similar reasoning also applies to relevant products of the MEP pathway, such as the volatile isoprene and the non-volatile xanthophyll, zeaxanthin. Isoprene and zeaxanthin may both increase the rigidity of thylakoid membranes in high light-grown plants concomitantly exposed to heat and water stress (drought stress, *sensu stricto*), and preserve lipids membrane from peroxidation [90,91,92,93]: this is an antioxidant function, *sensu* Gutteridge and Halliwell [21]. Even though these “indirect” functions should be regarded as antioxidant, the vast majority of plant scientists commonly refer to more restrictive definitions of antioxidants. The very same reasoning also applies to the definition of “oxidative damage”, and Halliwell [94] posed the “debatable” question of direct or indirect biomolecular damage caused by an attack of ROS upon the constituents of living organisms [95,96]. 

Definitions delineate the frame, within which we have to reason to assess the effective antioxidant functions of any substance in an in planta condition. Therefore, any definition must be unbiased and robust, as much as possible. Foyer and Noctor [23] defined antioxidant as a compound that “outcompetes others in reacting with ROS to give a relatively stable oxidized product”. The authors specifically refer to the quenching/scavenging of ROS, and introduce the concept of effective antioxidant, partly using an older definition of antioxidant given in 1995 by Halliwell and Gutteridge: “any substance that, when present at low concentrations compared with those of an oxidizable substrate, significantly delays or prevents oxidation of that substrate” [97]. Hernandez et al. [22] reasoned on the relevance of flavonoids as an antioxidant in plants, and defined an antioxidant as “a molecule that donates electrons or hydrogen atoms, yields an antioxidant derived radical that is efficiently quenched by other electron or hydrogen sources to prevent cellular damage; and whose properties are spatially and temporally correlated with oxidative stress events”. This is a restrictive definition compared to those given by Gutteridge and Halliwell [21] and Foyer and Noctor [23]. Within the framework delineated by their definition, Hernandez et al. [22] posed serious concerns on the relevance of flavonoids as antioxidants in an in planta situation, since flavonoid radicals have not been detected in the vast majority of studies. We note, however, that by just following the authors’ definition, antioxidant-derived radicals are to be efficiently reduced to prevent oxidative/cellular damage. It is conceivable, therefore, that the phenoxyl radicals, the products of flavonoid oxidation, are at the trace level in healthy cells and unlikely detectable even with the most sensitive equipment [31]. In actuality, Hernandez et al. [98] detected quinones (i.e., the phenoxyl radicals ECQ and EGCGQ) of both epicatechin (EC) and epigallo catechin gallate (EGCG) (the reduced flavonoid forms) in tea plants exposed to severe drought stress, when relative water content (RWC) dropped down below 60% and maximum PSII quantum yield for photochemistry, F_v_/F_m_, was as low as 0.5. It is likely this study was not the most appropriate to unveil the actual antioxidant functions of flavonoids in an in planta situation, since the concentration of ECQ was 25 times higher than that of EC, and EGCGQ and EGCG did not differ much in concentration. We reason that either (perhaps both) the reduction system of flavanols was severely compromised and/or, more probably, damage to the vacuolar membrane occurred, under the severe drought stress event plants were faced with. We therefore suggest that the detection of flavonoid radicals is not the best-suited estimate of their occurred antioxidant action. 

The required spatio-temporal correlation between antioxidants and oxidative stress events proposed by Hernandez et al. [22] also poses further questions. For instance, products synthesized through the plastidial MEP pathway, such as β-carotene and isoprene, have been reported as actual quenchers of singlet oxygen, based upon the generation of their oxidation products [99,100,101,102,103,104,105]. Since the production of isoprene and its oxidation products (methyl vinyl ketone and metacrolein) decreases under prolonged stress conditions [93,106,107,108,109,110] unlikely may accomplish increased antioxidant protection when oxidative stress becomes more severe. Similarly, the concentrations of a suite of β-carotene endo-peroxides and of the volatile β-cycocitral generated by singlet oxygen-induced oxidation of β-carotene, decline as light stress become particularly severe [101,102]. Similar observations have been also reported in the case of well-recognized antioxidants, such as antioxidant enzymes and ascorbate. There is vast and compelling evidence indeed that the activity of antioxidant enzymes may steeply decline during stress progression, when their action should be at the maximum [111,112,113,114,115]. This is the case of plants suffering from high temperature or drought stress when concomitantly exposed to high solar irradiance, as also recently shown for the large declines in antioxidant enzyme activities in *Platanus × acerifolia* leaves during the hottest hours of the day [93]. There is consensus indeed, that “stress-sensitive” species or genotypes are unable to maintain an efficient system for ROS removal when long exposed to stress [116]. This is because large restrictions in the plant’s ability to use radiant energy for carbon fixation, translates into severe excess light stress antioxidant enzymes are unable to counter efficiently [112,117,118,119]. 

There is also evidence that the level of ascorbate (ASC) declines markedly, as also observed for the activity of ascorbate peroxidase, in the chloroplast, while steeply increasing in the vacuole because of both high light and drought stress [120,121]. This stress-induced subcellular ASC redistribution decreases the usually (under non-stress conditions) high reducing the capacity of the chloroplast stroma [23]. This poses some concerns on the effective antioxidant role of ASC (based on the spatio-temporal relationship between oxidative stress event and proper antioxidant location, [22]) when the excess of radiant energy becomes particularly severe. There is old evidence that ASC is a relatively poor substrate [122,123] for vacuolar peroxidase aimed at reducing H_2_O_2_ likely entering the vacuole at considerable rates under the most severe stressful conditions [117,124,125,126,127]. Notably, the vacuole is the preferential site of flavonoid accumulation, although in some species colorless flavonoids have been detected in the chloroplast and in the nucleus. The functional significance of chloroplast- and nuclear-located flavonoids in preserving cells from irreversible oxidative damage has been already explored [16,31], and it will be briefly discussed in the next section. In contrast to ASC, flavonoids are excellent substrates for vacuolar class III peroxidases (POX), [122,125,128]. More than two decades ago, Yamasaki et al. [128] proposed a model in which phenoxyl radicals generated by the action of POX are recycled back to their reduced forms by ascorbate, and by mono-dehydroascorbate reductase as well, which act as “secondary antioxidants” [123,129]. At that time, the model assumed an exclusive distribution of flavonoids in the vacuole of epidermal cells, thus raising the question about how H_2_O_2_ generated in mesophyll cells might reach that compartment. There is now compelling evidence that H_2_O_2_ is transported at both intra- and inter-cellular level [130,131,132,133] through the action of the very same H_2_O transporters: H_2_O_2_ and H_2_O have indeed very similar physico-chemical properties [133,134,135,136]. There is evidence that aquaporins are involved indeed in oxidative stress responses [136,137].

In addition, we note that the epidermis is not the tissue with the highest flavonoid level in plants living under natural conditions. The concentration of epidermal flavonoids, usually estimated by fluorescence microscopy of Naturstoff reagent-stained tissues, largely exceeds that in mesophyll tissues, simply because of the small volume in which epidermal flavonoids are dissolved (Figure 3). The whole-leaf content of flavonoids may reach as much as 8-10 micromoles in plants long-exposed to full sunlight [17,18], but less than 1 micro mole may accumulate in the vacuoles of epidermal layers, because of their relatively low solubility in the aqueous cellular milieu [138]. Flavonoids located in the vacuoles of mesophyll cells are therefore close enough to ROS generating organelles (Figure 3), and hence optimally suited to buffer large alterations from ROS homeostasis. Furthermore, the view that flavonoids must be located in the very same cell organelle of ROS generation is likely to be strongly re-thought: ROS are not only involved cell-to-cell [132,139], but also in leaf-to-leaf communications, as is the case of light-induced control of stomata aperture [140,141]. There is also intriguing evidence that collapsed chloroplasts are included in the vacuole following both UV-B and high light stress (so-called chlorophagy) [142,143], as usually occurs in response to broad oxidative stress conditions [144,145,146]. We likely have to re-draw the picture representing the complexity of the dynamic cellular organization during the most severe stressful conditions, to delineate the actual scenario within which biological processes occur. Autophagy is functional in ROS detoxification indeed [147], and there is a possibility that flavonoids might be involved in this process. 

**Figure 4:**
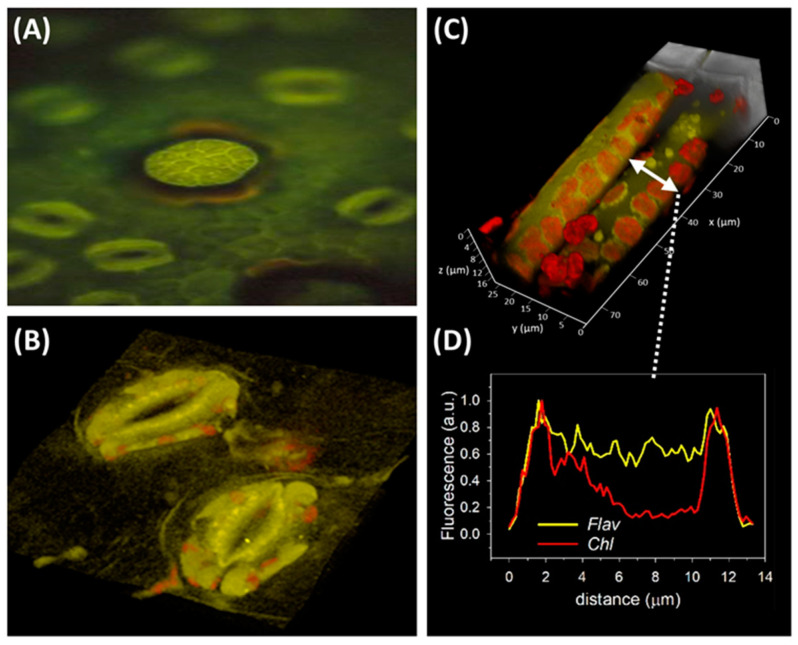
Flavonoids occur in stomata guard cells and chloroplasts. (**A**) Fluorescence image of Naturstoff-stained abaxial leaf surface excited at 365 nm and fluorescence image acquired at 580 nm. (**B**). Confocal laser scanning (CLS) 3D image showing flavonoids (yellow fluorescence recorded over 562–646 nm waveband) and chlorophyll (red fluorescence recorded over the 687–757 nm waveband), respectively, in stomata guard cells under 488 nm excitation. Distribution of yellow fluorescence is consistent with a cytoplasmic location of flavonoids (**C**) A CLS 3D view of palisade mesophyll cells showing the distribution of flavonoids in the vacuole and in the chloroplasts. (**D**) Profiles of fluorescence intensity of chlorophyll and flavonoids throughout the entire cell as shown by the arrow in (**C**), showing the co-localization of chlorophyll and flavonoid fluorescence.

Recent findings from Gloria Muday’s lab have offered conclusive support for an effective role of flavonoids as in vivo scavengers of H_2_O_2_ (for a review, see Chapman et al. [148]). In both *Arabidopsis* and tomato, flavonols, particularly quercetin mostly occurring in the cytoplasm and in the nucleus, greatly reduced the levels of H_2_O_2_ (estimated by fluorescence microscopy), thereby antagonizing ABA-induced stomatal closure [149,150]. Flavonols were also effective in preserving the integrity and the growth of pollen tubes upon heat stress in tomato, by decreasing ROS levels [151]. Agati et al., 2007 [152], also offered compelling evidence for the effective ability of quercetin (and luteolin) derivatives to quench singlet oxygen (^1^O_2_) generated by high PAR irradiance using cross-sections of *P. latifolia* leaves with largely different levels of flavonoids. It was shown that dihydroxy B-ring-substituted flavonoids accumulated in the chloroplast outer envelope membrane and, hence optimally suited to quench ^1^O_2_ (Figure 4).

We observe that albeit the formation of flavonoid oxidation products is conclusive proof of their occurred in vivo antioxidant actions, the very same strong evidence comes from their capacity to reduce the level of oxidative stress. We suggest that the reduction of oxidative stress may be well suited to estimate the antioxidant role of flavonoids in an in planta situation. As already noted the occurrence of flavonoid oxidation products may be transient and largely dependent upon the severity of stress indeed, and hence, unlikely suitable for systemic measurements. Instead, newly developed imaging techniques seem to be suitable for quantifying ROS, and hence oxidative stress, not only at the cell and organ [153], but also at the whole-plant level [154,155]. We believe that a plant antioxidant is best defined in terms of its ability to “counter” oxidative stress and damage: this ability is suitably “quantified” by measurements of “oxidative stress reduction” more than by the formation of oxidation products. 

## 4. Conclusions: Still Open Questions

In the previous section, we offered compelling evidence of in vivo ROS scavenging by “cytoplasm-located” flavonoids (Figure 4 A–D) [149,150,151,152]. Nonetheless, there are very few examples of this subcellular flavonoid distribution, which also includes nuclear flavonoids [31,156,157]. The potential antioxidant effects of nuclear-located flavonoids are of the greatest significance, as they include not only ROS quenching, but also the ability of antioxidant flavonol glycosides to chelate transition metal ions, thereby limiting the generation of ROS: this has to be regarded as an antioxidant function *sensu lato*. Nonetheless, studies aimed at exploring the sub-cellular distribution of flavonoids have not increased much in number over the last few years, and future investigation aimed at extending our knowledge on the sub-cellular distribution of specific flavonoid classes may help to solve the matter. 

That said, the discussion about the effective antioxidant role of flavonoids in plants has been (and is still) based upon their vacuolar distribution, as observed in the vast majority of plants. In turn, this imposes to reason about the contribution of the H_2_O_2_-scavenging by vacuolar located flavonoids in modulating stress-induced changes in whole-cell redox homeostasis. The very same reasoning applies to the actual contribution of the vacuole in the maintenance of cellular redox homeostasis under stressful conditions, e.g., during excessive light [153,158,159]. There are suggestions, indeed, that H_2_O_2_ may enter the vacuole at low rates to have major significance in altering the whole-cell redox homeostasis [22]. We note that the rates at which H_2_O_2_ may diffuse out of the chloroplast (or the peroxisome) [117,125,160,161] when the activity and the concentration of primary antioxidants decline greatly in response to severe stress are practically impossible to quantify. Additionally, we observe that the concentration range within which H_2_O_2_ activates signaling pathways to enhance further stress resistance or instead irreversibly damages cell metabolism and functioning appears very narrow [23,162]. We highlight methods used to quantify H_2_O_2_ are conducted at the level of the whole-organ and, hence, are unsuitable to detect the small changes in H_2_O_2_ concentration that may occur in a very short time level in different subcellular compartments, and are responsible for the subsequent cell fate. Our deterministic approach encounters, once again, insurmountable scale-level obstacles.

In our opinion, the conclusive question regards the role of flavonoids relative to that of the modular and integrated network of antioxidant defenses plants activate in response to stress conditions of increasing severity. Plants face photo-oxidative stress of increased severity not only on long- (days/weeks) but also on a short-term (hour) basis. For instance, solar irradiance is in a large excess of that used for photosynthesis during the central hours of the day (midday depression of photosynthesis), because of stomatal limitations and supra-optimal leaf temperatures [163]. This, in turn, leads to reductions in both Rubisco activation, thereby imposing additional biochemical limitations to photosynthesis [164,165,166] and PSII photochemical efficiency. As already noted, excess excitation energy-induced large alterations in redox homeostasis are the very conditions that depress the activity of primary antioxidants [107,167,168,169], while promoting the biosynthesis of antioxidant flavonoids [19,93,113,170]. Unveiling the extent to which primary and secondary antioxidants vary, on a short-term basis, in response to abrupt changes in excess radiant energy may help to solve the question of the actual significance of flavonoids in the modular and integrated network of antioxidant defenses. The notion that large alterations in the cell redox state trigger the biosynthesis of flavonoids [72,171] leads us to hypothesize that flavonoids may complement the functions of primary antioxidants when their ROS detoxifying capacity declines in plants suffering from severe photooxidative stress [93,112,113,170]. The matter needs future investigation aimed at estimating the changes in the whole spectrum of primary and secondary antioxidant defenses in plants with constitutively different levels of antioxidant flavonoids (using both natural or induced variation), challenged against the excess of radiant energy of increasing severity induced by abiotic stressors of different origin.
